# Risk Stratification and Treatment Algorithm of Metastatic Renal Cell Carcinoma

**DOI:** 10.3390/jcm10225339

**Published:** 2021-11-16

**Authors:** Marc-Oliver Grimm, Katharina Leucht, Susan Foller

**Affiliations:** Department of Urology, Jena University Hospital, Friedrich-Schiller University, 7743 Jena, Germany; katharina.leucht@med.uni-jena.de (K.L.); susan.foller@med.uni-jena.de (S.F.)

**Keywords:** renal cell carcinoma, immune-checkpoint inhibitors, axitinib, avelumab, nivolumab, ipilimumab, pembrolizumab, cabozantinib

## Abstract

Systemic therapy for metastatic renal cell carcinoma has continuously evolved over the last two decades. Significant improvements in overall survival and quality of life of patients with advanced disease have been observed. With the approval of combination therapies with PD(L)-1 immune checkpoint inhibitors (ICI) as first-line therapy in 2019, the previous standard VEGFR-TKI monotherapy has been replaced as the primary treatment option. In addition to immunotherapy with nivolumab and ipilimumab, three VEGFR-TKI/ICI combinations are now approved. Therapy selection should be preceded by risk stratification using defined criteria from the International Metastatic Renal Cell Carcinoma Database Consortium (IMDC). Clinical parameters, as well as detailed patient counseling on differences in the efficacy profile (response rate, long-term progression-free survival), potential side effects, and impact on quality of life, are of key importance in the individual treatment decision.

## 1. Introduction

Renal cell carcinoma (RCC) accounts for approximately 90% of all malignant kidney diseases. They are classified into different histologic subtypes with the clear cell variant being most frequent (80–90%). Papillary and chromophobe carcinoma dominate the non-clear cell histologies with an overall proportion of 10–15% and 4–5%, respectively [1].

At initial diagnosis, 20–30% of RCCs are metastatic, while another 20–30% of primarily localized tumors metastasize or develop local recurrence despite initial surgical treatment with curative intent [2].

**Figure 1:**
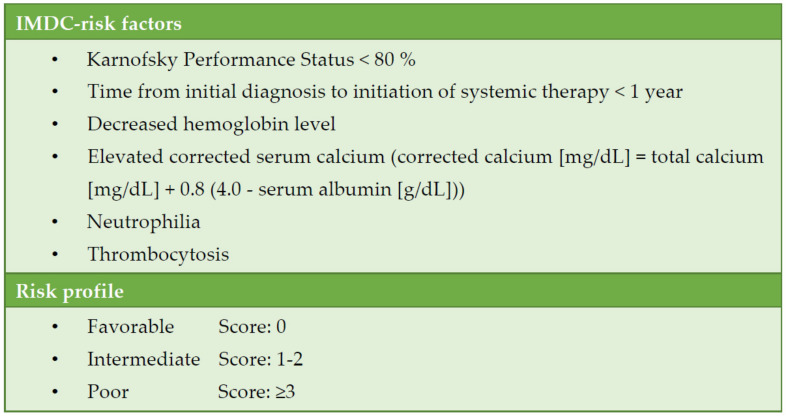
Risk assessment for metastatic renal cell carcinoma according to the International Metastatic RCC Database Consortium (IMDC).

Systemic therapy for metastatic RCC (mRCC) has continuously evolved over the past two decades. A patient’s individual risk for progression and death can be estimated according to the International Metastatic Renal Cell Carcinoma Database Consortium (IMDC) (Figure 1). Classification into favourable, intermediate, and high IMDC risk strongly correlates with survival time and is the basis for treatment selection [3]. 

In the last decade, tyrosine kinase inhibitors directed against the vascular endothelial growth factor receptor (VEGFR-TKIs) were by far the most frequently applied therapy. This changed fundamentally in 2019 with the approval of combination therapies, each of which includes a PD-(L)1 immune checkpoint inhibitor (ICI) as the new treatment backbone. Currently, dual checkpoint inhibition by a combination of the PD-1 inhibitor nivolumab and the inhibitor of the cytotoxic T-lymphocyte-associated protein 4 (CTLA-4) ipilimumab as well as three VEGFR-TKI/ICI combinations are approved; an additional approval is expected this year.

## 2. First-Line Therapy

In the respective pivotal trials, the approved ICI combinations were compared with a former first-line standard, the VEGFR-TKI sunitinib. The combination therapies examined were:

- nivolumab + ipilimumab (CheckMate 214 [4]);
- pembrolizumab + axitinib (Keynote 426 [5]);
- avelumab + axitinib (JAVELIN renal 101 [6]);
- nivolumab + cabozantinib (CheckMate 9ER [7]).

**Figure 2:**
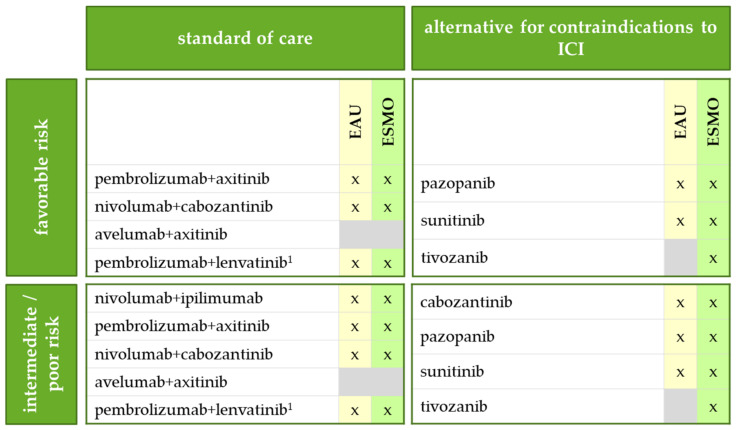
First-line therapy for metastatic renal cell carcinoma (RCC) depending on the risk profile according to the International Metastatic RCC Database Consortium (IMDC), based on EAU [8] and ESMO guidelines [9] for RCC. Gray: no recommendation. ^1^: Not yet approved by the EMA. ICI, immune-checkpoint inhibitor.

Except for nivolumab + ipilimumab, which is approved only for patients with intermediate and poor-risk IMDC risk, the combinations are approved for patients of all risk groups (Figure 2). In this regard, the guidelines of the European Association of Urology (EAU) for renal cell carcinoma and the European Society for Medical Oncology (ESMO) recommend pembrolizumab + axitinib and nivolumab + cabozantinib regardless of IMDC risk profile as well as nivolumab + ipilimumab for intermediate and poor risk [8,9]. Although approved by the European Medicines Agency (EMA), avelumab + axitinib has not yet been recommended (except for the german guideline), as an advantage over sunitinib regarding overall survival has not been observed so far (see below). Additionally, the EAU has already recommended another ICI/VEGFR-TKI combination, pembrolizumab + lenvatinib, which has provided promising results in the pivotal clinical trial CLEAR [8]. However, the combination has not yet been approved by the EMA.

### 2.1. Nivolumab + Ipilimumab (CheckMate 214)

The co-primary endpoints of CheckMate 214 were objective response rate (ORR), progression-free survival (PFS), and overall survival (OS) in patients with intermediate and poor IMDC risk profiles. Long-term data are available with a minimum follow-up of five years. Significant benefits in OS have been reported, as, for intermediate/poor risk, 5-year-survival rates were 43% for the combination therapy, as compared to 31% for sunitinib. For nivolumab + ipilimumab the PFS curve showed a plateau beginning at 30 months and corresponding to long-term remissions or stabilizations in 31% of patients after five years (intermediate/poor risk). For intermediate/poor risk also ORR was significantly increased for the combination (42% vs. 27%, Table 1), with 11% of mostly long-lasting complete remissions (CR; 11% vs. 2%) [10]. The rate of primary progressive patients has not yet been reported for the five-year follow-up but was 19% after four years of minimum follow-up (vs. 17% for sunitinib) [11] For favorable risk, a limited number of OS-related events have been observed so far. ORR and PFS are numerically in favor of sunitinib in this risk group while OS-curves overlap. Accordingly, the nivolumab + ipilimumab is not approved for these patients. However, also for patients with favorable risk, plateauing in the duration of response and PFS is evident in the long-term follow-up with potential impact on OS in the long run [10].

In CheckMate 214, PD-L1 tumor cell expression has been studied as a biomarker in patients with intermediate and poor risk. PD-L1-positive patients appear to benefit most from combination therapy (PFS hazard ratio (HR) 0.46 (95% CI 0.31–0.67); OS HR 0.45 (95% CI 0.29–0.71)). In PD-L1-negative patients, an advantage is found in OS (HR 0.73 (95% CI 0.56–0.96)), but not for PFS [4].

### 2.2. Pembrolizumab + Axitinib (Keynote 426)

The co-primary endpoints of the Keynote 426 study were OS and PFS in the overall cohort. For pembrolizumab + axitinib, there was a significant improvement in PFS (HR 0.68) and OS (HR 0.73) after a median follow-up of 43 months (Table 1). The ORR was 60% (vs. 40% for sunitinib); the CR rate was 10% (vs. 3.5%). Of note, the disease control rate was high at 83% (vs. 75%); only 11.3% of the patients were primary progressive (vs. 17.0%).

Subgroup analyses revealed that the benefits of pembrolizumab + axitinib in terms of PFS and OS are limited to intermediate and poor-risk patients, whereas patients with favorable risk do not seem to benefit vs. sunitinib. No plateau formation in the PFS curve designating long-term remissions as reported for nivolumab + ipilimumab is seen even with longer follow-up [12]. PD-L1 expression seems to be without prognostic relevance.

### 2.3. Avelumab + Axitinib (JAVELIN Renal 101)

The co-primary endpoints of the JAVELIN renal 101 study were PFS and OS in PD-L1 positive tumors. After 19 months of median follow-up, the study showed a significant improvement in PFS in the PD-L1-positive group (HR 0.62) as well as for the overall population (HR 0.69, Table 1). For OS, the data are immature. To date, with an HR of 0.80 for the overall population, no significant benefit has been demonstrated for the avelumab + axitinib combination. ORR was 56% for PD-L1-positive tumors (vs. 27% for sunitinib) with 5.6% CR (vs. 2.4%). The disease control rate was 83% for the combination vs. 69% for sunitinib. Of the patients, 12 vs. 22 were primary progressive [13].

Subgroup analyses suggest advantages for the combination therapy over sunitinib in all relevant subgroups; again, the biomarker PD-L1 showed no correlation with response [13].

### 2.4. Nivolumab + Cabozantinib (CheckMate 9ER)

The randomised phase 3 trial CheckMate 9ER evaluated nivolumab in combination with cabozantinib in mRCC. Nivolumab was administered at standard dose, cabozantinib at a reduced oral dose of 40mg/day instead of 60mg/day, usually applied as a monotherapy. The comparator in the standard arm was sunitinib in the standard regimen. The primary endpoint was PFS. After a median follow-up of 23.5 months, median PFS was almost twice as long with the combination as with sunitinib (HR 0.52, Table 1). All IMDC risk groups appeared to benefit, with the strongest effect in poor-risk patients (HR 0.36) and the smallest effect for favorable risk (HR 0.58). According to subgroup analysis, PD-L1 status was not prognostically relevant. There was also a significant benefit in OS (HR 0.66). Of note, the ORR was 55% for the combination as compared to 28% for sunitinib, with 9.3% and 4.3% of patients with CR, respectively. Very few patients were primary progressive (6.2% vs. 14%), and the disease control rate was 88% (vs. 70%) [7,14,15].

### 2.5. Pembrolizumab + Lenvatinib (CLEAR)

Further promising results were recently reported for the 3-arm CLEAR study. Study arms were (i) pembrolizumab (200mg every three weeks, i.v.) + lenvatinib (20mg/day, oral), (ii) lenvatinib (18mg/day, oral) + everolimus (5mg/day, oral), and (iii) sunitinib (standard regimen). The primary endpoint was PFS. After a median follow-up of 27 months, the median PFS was 24 months for pembrolizumab + lenvatinib vs. 9.2 months for sunitinib (HR 0.39, Table 1). Patients benefited from pembrolizumab + lenvatinib regardless of IMDC risk profile and PD-L1 status. Median OS has not been reached in any of the study arms to date, but pembrolizumab + lenvatinib (in contrast to lenvatinib + everolimus) showed advantages over sunitinib (HR 0.66). ORR for pembrolizumab + lenvatinib was remarkable with 71% (vs. 36% for sunitinib), with 16% CR. Disease control rate was 90% for the combination vs. 74% for sunitinib. Of the patients, 5.4% vs. 14% were primary progressive [16,17]. However, when (indirectly) comparing the pembrolizumab + lenvatinib efficacy data to those of other trials (e.g., CheckMate 9ER), it should be considered that CLEAR overall included patients with a more favorable IMDC risk profile.

## 3. Second-Line Therapy

The approval of PD-(L)1-ICI in first-line therapy influences the therapeutic options after disease progression.

No standard has been established after ICI-based combination therapy, but in principle, all known effective VEGFR-TKI (subject to the approval status and if not previously administered (axitinib, cabozantinib)) as well as the combination lenvatinib + everolimus can be considered [8,9]. Many experts favor cabozantinib due to its apparent advantages over sunitinib in first-line therapy and (limited) second-line data after PD-(L)1-ICI therapy [8,18]. Others prefer other modern VEGFR TKI, such as tivozanib, due to its perceived favorable side effect profile [19]. Furthermore, promising results of a phase 2 trial with pembrolizumab plus lenvatinib after progression upon PD-(L)1-targeted therapy are available (ORR: 52%, 12-months PFS: 44%, 12-months OS: 77% [20]), but, however, currently, the combination is not approved in this indication. In addition, observational second-line studies of known TKI are ongoing, as are studies with new agents (e.g., HIF-2α inhibitors).

For patients with a favorable risk profile receiving first-line VEGFR TKI-targeted therapy nivolumab or cabozantinib remain the standard second-line treatment options recommended by current guidelines. Other options include lenvatinib + everolimus and other VEGFR-TKI [8,9].

## 4. Side Effects

The new first-line combination therapy approaches (immune-immune vs. immune-TKI) differ with respect to the spectrum of side effects.

Axitinib, like cabozantinib and lenvatinib, is associated with known chronic VEGFR-TKI toxicity. Diarrhea, hypertension, fatigue, hypothyroidism, hand-foot syndrome (palmar-plantar erythrodysaesthesia syndrome), and gastrointestinal side effects like nausea, or loss of appetite are most common [21].

For ICI the central focus is on immune-mediated side effects. These mainly involve skin with exanthema or pruritus, lung with pneumonitis, intestine with diarrhea and/or colitis, liver with hepatitis and/or transaminitis, and endocrine system with hypo- or hyperthyroidism, hypophysitis, or adrenal insufficiency [22]. Immune-mediated side effects often manifest as nonspecific symptoms such as fatigue and gastrointestinal discomfort, which may become clinically apparent even after discontinuation of immunotherapy. In contrast to the chronic toxicity caused by VEGFR-TKI, immune-mediated side effects are predominantly transient. However, side effects on the endocrine system are often irreversible and may require permanent hormone replacement [23]. Patients should be informed about this risk prior to initiation of therapy.

For management of immune-mediated side effects, interruption or permanent discontinuation of ICI therapy are indicated. Dose reductions/modifications are not foreseen for ICI. In general, for grade 1 CTCAE immune-mediated side effects, therapy can usually be continued; for grade 2, therapy interruption plus corticosteroid administration is recommended; and for grade 4, permanent discontinuation of therapy with concomitant administration of systemic corticosteroids is advised [24]. Recommendations for the management of grade 3 immune-mediated adverse events are heterogeneous, partly interruption, partly discontinuation. If therapy was interrupted and immune-mediated side effects have decreased to at least grade 1 under corticosteroids, the latter should be slowly tapered over a period of four weeks. When the corticosteroid dose could be reduced to physiological levels (≤10 mg prednisolone or equivalent), therapy may be resumed.

When an ICI is combined with a VEGFR-TKI, the toxic effects appear to add up numerically. Among others, the differential diagnosis for hepatitis and diarrhea is crucial as, for these, an immune-mediated etiology can only be determined by excluding other causes. Overall, the combinations of ICI and VEGFR-TKI are well tolerated. In the respective pivotal trials high-dose corticosteroids were used as follows: pembrolizumab + axitinib: 27%, avelumab + axitinib: 11%, nivolumab + cabozantinib: 19%; pembrolizumab + lenvatinib: 15% (not approved yet), Table 2) [5,6,7,25]. For ICI/VEGFR-TKI combinations, the dosage of the VEGFR-TKI can be reduced to manage TKI-associated side effects, regardless of interruption of ICI therapy. In the case of axitinib, dosage may also be increased if well tolerated.

The ICI/ICI combination nivolumab + ipilimumab is associated with a high rate of immune-mediated side effects. These occur primarily during the first 12 weeks of therapy when both ICI are administered combinedly. 29% of patients received high-dose corticosteroids [11] and one in four patients discontinued therapy due to treatment-associated side effects (mainly transaminases increase, diarrhea, pneumonitis), mostly during combination doses. 79% of patients received all four intended combination doses. However, an analysis of the “discontinuers” suggests that these patients do not benefit any less from therapy in terms of ORR and OS [29]. Indeed, data suggest a relationship between (immune-mediated) side effects and treatment response. Thus, these patients probably even benefit most with “deep” responses, long-term remissions, or at least long treatment-free intervals.

## 5. Quality of Life

Quality of life (QoL) data are available for nivolumab + ipilimumab, nivolumab + cabozantinib, pembrolizumab + axitinib, and for the not yet approved combination pembrolizumab + lenvatinib (Table 3). QoL data are not yet available for axitinib + avelumab.

Patient-reported outcomes (PRO) from the questionnaires are influenced by drug-associated side effects on the one hand, and by changes in symptoms and quality of life due to treatment response on the other hand. In the described studies, questionnaires were used that preferentially consider disease symptoms (FACT-G (Functional Assessment of Cancer Therapy-General), FKSI-19 (FACT-Kidney Symptom Index), FKSI-DRS (FKSI-Disease-Related Symptoms)) or focus on the different quality of life aspects (EORTC QLQ-C30, EQ-5D-3L VAS (Visual Analogue Scale).

In order to estimate when a change in PRO is clinically relevant, values are defined for each questionnaire or (sub)scale that describes the minimal improvement or deterioration required for this. These are often expressed as minimally important difference or prespecified threshold units [34].

In the PRO analyses of the described studies, the cyclic administration of the comparator therapy with sunitinib (four weeks of daily dosing, two weeks off) is reflected in the health status, which complicates the interpretation of the data depending on the time of collection.

In CheckMate 9ER, predefined thresholds for clinical relevance for FKSI-19, FKSI-DRS, and EQ-5D-3L VAS were not met in either study arm. Only for sunitinib PRO fell below the threshold for EQ-5D-3L utility index at weeks 61, 67, and 85. Nivolumab + cabozantinib nevertheless showed statistical PRO benefits compared with sunitinib. With the combination, the FSKI-19 score remained at baseline levels after an initial decline and was thus significantly better compared with sunitinib (from week 13). Disease-related symptoms (FKSI-DRS) even improved from baseline (significantly better vs. sunitinib). The combination also showed significant benefits over sunitinib for EQ-5D-3L utility index and VAS score, although not at all time points. The former remained at baseline levels, while the latter improved compared with a baseline from approximately week 19. The time-to-confirmed-worsening of PRO was significantly prolonged for nivolumab + cabozantinib compared with sunitinib (FKSI-19: HR 0.64; FKSI-DRS: HR 0.62; EQ-5D-3L VAS: HR 0.71) [33].

In the Sunitinib arm of Keynote-426, the minimally important difference for clinically relevant worsening of PRO was met multiple times for QLQ-C30 and EQ-5D-3L VAS for data requisition at the end of a four-week treatment period. For the combination, PRO was slightly below baseline during the reported period (through week 30), but without clinical relevance. Statistically, no significant differences were found for pembrolizumab + axitinib versus sunitinib in disease-related symptoms (FKSI-DRS), overall health status (EQ-5D-3L VAS), and QoL (EORTC QLQ-C30 Global Health Status/QoL) [32]. However, a greater worsening from baseline to week 30 was observed in the EORTC QLQ-C30 diarrhea symptom scale for pembrolizumab + axitinib compared with sunitinib [35]. When looking at the time to confirm deterioration in the EQ-5D-3L VAS, there was no difference for pembrolizumab + axitinib vs. sunitinib (HR 1.12) [32]. In contrast, in the FKSI-DRS score, the time to first confirm deterioration was in favor of sunitinib (HR 1.44) [35].

PRO has also been published for the not-yet-approved combination pembrolizumab + lenvatinib, but no conclusions on clinical relevance were reported. The combination is statistically comparable to sunitinib in terms of disease-associated symptoms (FKSI-DRS), general health status (EQ-5D-3L VAS), and the EORTC QLQ-C30 QoL questionnaire. When looking at subscales of the EORTC QLQ-C30, pembrolizumab + lenvatinib is significantly superior to sunitinib in the physical subscore and in the symptom scales of fatigue, dyspnea, and constipation. Compared with baseline, PRO in the EORTC QLQ-C30 questionnaire at maximum remains at baseline level during the reported 46-week period. In contrast, with respect to time to first and definite deterioration of PRO, the combination was significantly superior vs. sunitinib in several symptoms and QoL scales (first deterioration: EQ-5D-3L VAS: HR 0.83; definite deterioration: FKSI-DRS: HR 0.70; EQ-5D-3L: HR 0.75; EQ-5D-3L VAS: HR 0.67; EORTC-QLQ-C30 Global Health Status/QoL: HR 0.60) [28].

Comprehensive PRO data (FKSI-19, FACT-G, EQ-5D-3L questionnaires) are also available for dual checkpoint inhibition with nivolumab + ipilimumab. FKSI-19 reveals significant improvements after the initial 4 nivolumab + ipilimumab dosings vs. sunitinib, which, however, fulfill the criteria of being clinically relevant from week 37 on. The EQ-5D-3L VAS questionnaire shows similar results with repeatedly clinically relevant improvement after week 49. No clinically relevant changes were reported for FACT-G and EQ-5D-3L Utility Index. Data suggest fewer symptoms and better QoL for nivolumab + ipilimumab compared with sunitinib. Indeed, QoL is relatively stable during the period of combination administration and then improves compared with baseline. Compared with sunitinib, there is a sustained improvement in nearly all QoL measures [31].

## 6. Expert Point of View—Criteria for the Choice of First-Line Therapy

With four (soon to be five) combination therapies and additional monotherapies available, the individual choice of therapy can be difficult. Based on the guideline recommendations and approval texts, the advantages and disadvantages of the different combinations will be elaborated in the following chapter from the authors’ point of view.

According to current guidelines, the IMDC risk score should first be determined in order to distinguish between favorable and intermediate/poor prognosis. Of note, with only one risk factor being present, the prognosis is at least intermediate.

For patients with favorable risk profiles, the approved ICI/VEGFR-TKI combinations (but not nivolumab + ipilimumab) can be considered. However, subgroup analyses do not show significant survival benefits over sunitinib, a VEGFR-TKI monotherapy. Molecular analyses suggest that tumors of the favorable risk group feature a so-called angiogenesis profile, i.e., they respond relatively well to VEGFR-TKI. Due to the predominantly slow disease progression, sequential therapy can be considered for this risk group (e.g., VEGFR-TKI followed by nivolumab). Arguments in favor of first-line combination therapy are the higher ORR and possible advantages in PFS. This is opposed by a higher rate of side effects. From the authors’ point of view, sequential therapy should be mainly considered for patients of older age or with limited life expectancy due to comorbidities.

For intermediate/poor risk groups, ICI/VEGFR-TKI combinations as well as “pure” immunotherapy with nivolumab + ipilimumab can be considered. With the latter, chances for long-term remission (31% after five years), the absence of “chronic” side effects, and the quality-of-life data should be emphasized. Disadvantages are the lower disease control rate (73%) and the higher rate of immune-mediated side effects compared with ICI/VEGFR-TKI combinations. In the pivotal trial, these immune-related side effects led to discontinuation of therapy in about one out of four of patients. Provided the immune-mediated side effect healing without sequelae, these patients, however, benefit particularly, as further tumor therapy can often be avoided, at least for a longer period of time [11].

The main arguments in favor of ICI/VEGFR-TKI combinations are the high response and disease control rates. The latter seems to be particularly higher for combinations comprising “modern” TKI (cabozantinib, lenvatinib). Long-term data are available only for pembrolizumab + axitinib, not revealing long-term remissions as observed in the PFS curve with nivolumab + ipilimumab. In terms of QoL, there appear to be slight advantages for nivolumab + cabozantinib among the ICI/VEGFR-TKI combinations; in contrast, pembrolizumab + lenvatinib appears to achieve the highest tumor control (ORR, PFS).

From the authors’ point of view, ICI/VEGFR-TKI combinations should be especially given preference in patients with a high tumor burden. Younger patients with low to moderate tumor burden may benefit from nivolumab + ipilimumab, especially in the long term. This seems to be particularly true for patients with PD-L1-positive tumors (cut-off >1% positive tumor cells), in whom an ORR of 58% was initially reported for intermediate/poor risk, including 16% complete responses [4].

## 7. Key Points for Clinical Practice

- PD-(L)1 ICI are the new backbone of first-line therapy in mRCC;
- Nivolumab + ipilimumab is a standard first-line therapy in patients with intermediate and poor IMDC risk;
- Pembrolizumab + axitinib, nivolumab + cabozantinib, and avelumab + axitinib are standard first-line therapy regimens, regardless of the risk profile;
- For patients with favorable risk, VEGFR-TKI monotherapy may be considered as an alternative to combination therapy; in this case, cabozantinib or nivolumab remain standard second-line options;
- Patients should be comprehensively counseled about the advantages and disadvantages of the different combinations (immune-immune vs. immune-VEGFR-TKI), taking into account the individual situation and aims of treatment;
- Patient information should include survival benefit, remission rate, long-term remissions, side effects (“chronic” TKI side effects, risk of immune-mediated side effects), and quality of life.

## Figures and Tables

**Table 1:** Results of pivotal trials for combination therapies with PD-(L)1 immune checkpoint inhibitors (vs. sunitinib) for first-line treatment of advanced or metastatic renal cell carcinoma plus newly published data pembrolizumab + lenvatinib.

Study/ Treatment Arms	Primary End Points	Follow-Up (Months)	IMDC Risk	No. of Patients	ORR (%)	PFS (Months)	OS (Months)
CheckMate 214 [10,11] nivolumab + ipilimumab vs. sunitinib	ORR, PFS, OS (intermediate and poor risk)	68 (median)	ITT	1096	39 vs. 32 *p* = 0.0134	12 vs. 12 HR 0.86 (0.73–1.01) *p* = 0.0628	56 vs. 38 HR 0.72 (0.62–0.85) *p* < 0.0001
			intermediate and poor	847	42 vs. 27 *p* < 0.0001	12 vs. 8.3 HR 0.73 (0.61–0.87) *p* = 0.0004	47 vs. 27 HR 0.68 (0.58–0. 81) *p* < 0.0001
			favourable	249	30 vs. 52 *p* = 0.0005	12 vs. 29 HR 1.60 (1.13–2.26) *p* = 0.0073	74 vs. 68 HR 0.94 (0.65–1.37) *p* = 0.7673
Keynote 426 [12] pembrolizumab + axitinib vs. sunitinib	PFS, OS (ITT)	43 (median)	ITT	861	60 vs. 40 *p* < 0.0001	16 vs. 11 HR 0.68 (0.58–0.80) *p* < 0.0001	46 vs. 40 HR 0.73 (0.60–0.88) *p* < 0.001
			intermediate and poor	592	57 vs. 35	14 vs. 8.2 HR 0.67 (0.55–0.81)	NE vs. 29 HR 0.64 (0.52–0.80)
			favourable	269	69 vs. 50	21 vs. 18 HR 0.76 (0.56–1.03)	NE vs. NE HR 1.17 (0.76–1.80)
JAVELIN renal 101 [13] avelumab + axitinib vs. sunitinib	PFS, OS (PD-L1 (+))	19 (median)	ITT	886	53 vs. 27	13 vs. 8.0 HR 0.69 (0.57–0.83) *p* < 0.0001	NE vs. NE HR 0.80 (0.62–1.03) n.s.
			PD-L1 (+)	560	56 vs. 27	14 vs. 7.0 HR 0.62 (0.49–0.78) *p* < 0.0001	NE vs. 29 HR 0.83 (0.60–1.15) n.s.
CheckMate 9ER [14] nivolumab + cabozantinib vs. sunitinib	PFS (ITT)	24 (median)	ITT	651	55 vs. 28	17.0 vs. 8.3 HR 0.52 (0.43–0.64) *p* < 0.0001	NE vs. 29.5 HR 0.66 (0.50–0.87) *p* = 0.0034
			poor	129	38 vs. 10	9.9 vs. 4.2 HR 0.36 (0.23–0.56)	NE vs. 11 HR 0.45 (0.27–0.76)
			intermediate	376	56 vs. 29	18 vs. 8.5 HR 0.58 (0.45–0.76)	NE vs. NE HR 0.74 (0.50–1.08)
			favourable	146	66 vs. 44	25 vs. 13 HR 0.58 (0.36–0.93)	NE vs. NE HR 0.94 (0.46–1.92)
CLEAR [16,17] pembrolizumab + lenvatinib vs. sunitinib	PFS (ITT)	27 (median)	ITT	712	71 vs. 36 (*p* < 0.001)	24 vs. 9.2 HR 0.39 (0.32–0.49) *p* < 0.001	NE vs. NE HR 0.66 (0.49–0.88) *p* = 0.005
			intermediate and poor	271	72 vs. 66	22 vs. 5.9 HR 0.36 (0.28–0.47)	NE vs. NE HR 0.58 (0.42–0.80)
			favourable	226	68 vs. 51	28 vs. 13 HR 0.41 (0.28–0.62)	NE vs. NE 1.15 (0.55–2.4)

HR, hazard ratio; ORR, objective response rate; PFS, progression-free survival; OS, overall survival; NE, not evaluable; ITT, intention-to-treat, n.a., not available; n.s., not significant.

**Table 2:** Rate of serious adverse events with PD-(L)1-ICI-based combination therapies. (tr)AE, (treatment-related) adverse events.

		trAE Grade ≥3 (%)	Treatment- Related Deaths (%)	AE-Associated Discontinuation (%)	Prednisone ≥ 40 mg (%)
CheckMate 214 [11]	ipilimumab + nivolumab	49	1.5 ^1^	23 **	29
Keynote 426 [5,26]	pembrolizumab + axitinib	67	0.9 ^2^	31 */11 **	27
JAVELIN renal 101 [6,27]	avelumab + axitinib	57	0.7 ^3^	23 */7.6 **	11
CheckMate 9ER [7]	nivolumab + cabozantinib	61	0.3 ^4^	15 */3.1 **	19
CLEAR [17,25,28]	pembrolizumab + lenvatinib	72	1.1 ^5^	37 */13 **	15

^1^ pneumonitis (*n* = 1), pneumonia + aplastic anemia (*n* = 1), immune-mediated bronchitis (*n* = 1), liver toxic effects (*n* = 1), lower gastrointestinal hemorrhage, (*n* = 1), sudden death (*n* = 1), hemophagocytic syndrome (*n* = 1), lung infection (*n* = 1); ^2^ myasthenia gravis (*n* = 1), myocarditis (*n* = 1), necrotizing fasciitis (*n* = 1), pneumonitis (*n* = 1); ^3^ myocarditis (*n* = 1), necrotizing pancreatitis (*n* = 1), sudden death (*n* = 1); ^4^ small-intestine perforation (*n* = 1); ^5^ acute renal failure (*n* = 1), uncontrolled hypertension (*n* = 1), complications from myasthenic syndrome (*n* = 1), complications from autoimmune hepatitis (*n* = 1). * discontinuation of at least one drug. ** discontinuation of both drugs. AE, adverse events; trAE, treatment-related adverse events.

**Table 3:** Patient-reported outcomes (PRO) and quality of life (QoL). Adapted from [30].

	CheckMate 214 [31]		Keynote 426 [32]		CheckMate 9ER [33]		CLEAR [28]	
	Nivolumab + Ipilimumab		Pembrolizumab + Axitinib		Nivolumab + Cabozantinib		Pembrolizumab + Lenvatinib	
	Up to Week 103		Week 30		Up to Week 91		Week 46	
	vs. Sunitinib	vs. Baseline	vs. Sunitinib	vs. Baseline	vs. Sunitinib	vs. Baseline	vs. Sunitinib	vs. Baseline
FKSI-19	+	+			+	=		
FKSI-DRS			=	(−)	+	(+)	=	
FACT-G	+	(+)						
EQ-5D-3L VAS	=	(+)	=	(−)	+	(+)	=	
EQ-5D-3L utility index	+	(+)			+	=		
EORTC QLQ-C30			= ^1^	(−)			= ^2^	=

FACT-G, Functional Assessment of Cancer Therapy-General; FKSI-19, FACT-Kidney Symptom Index; FKSI-DRS, FKSI-Disease-Related Symptoms; EORTC QLQ-C30, European Organization for Research and Treatment of Cancer (EORTC) Quality of Life Questionnaire-C30; EQ-5D-3L VAS, Questionnaire from the EuroQol Group (5 dimensions, 3 levels). + PRO/QoL with combination therapy statistically significantly better (vs. sunitinib) or clinically relevantly better (vs. baseline), respectively; (+) PRO/QoL with combination therapy numerically better (vs. baseline) without clinical relevance; (−) PRO/QoL with combination therapy numerically worse (vs. baseline) without clinical relevance; = no statistically significant difference (vs. sunitinib) or no numerical difference (vs. baseline). ^1^ global health status only. ^2^ functional scale: physical subscore: significant in favor of pembrolizumab + lenvatinib. symptom scale: fatigue, dyspnoea, obstipation: significant in favor of pembrolizumab + lenvatinib.
